# Distal radial artery cannulation in the Hegu (LI4) region for perioperative invasive arterial blood pressure monitoring: two case reports

**DOI:** 10.3389/fmed.2026.1929045

**Published:** 2026-08-07

**Authors:** Long Zhang, Shunli Diao, Jianlin Wang, Yingying Liu, Jie Chen, Liangguang Zhang

**Affiliations:** 1 Department of Anesthesiology, Ningbo No. 6 Hospital, Ningbo, Zhejiang, China; 2 Ningbo Clinical Research Center for Orthopedics, Sports Medicine and Rehabilitation, Ningbo, Zhejiang, China

**Keywords:** case report, distal radial artery, Hegu region, invasive arterial blood pressure monitoring, ultrasound guidance

## Abstract

**Background:**

Invasive arterial blood pressure monitoring is essential for perioperative hemodynamic management. While proximal radial artery cannulation is widely used due to its ease and low complication rate, it is unsuitable for patients with upper-limb trauma, wrist/forearm surgery, local soft-tissue injury, or prior vascular injury from puncture. Distal radial artery access at the Hegu (LI4) region—originally developed for cardiovascular procedures—offers a viable alternative: it lies superficially, is less affected by wrist motion, and benefits from collateral perfusion via the ulnar artery and palmar arches.

**Case presentation:**

Two patients required alternative arterial access due to limited conventional proximal radial artery access. Case 1: a 41-year-old woman after emergency right upper-limb replantation for traumatic amputation. Case 2: a 50-year-old man after debridement, internal fixation, and free anterolateral thigh flap reconstruction for a severe right-hand crush injury. In both, the left distal radial artery (Hegu region) was used for invasive arterial pressure monitoring. Under local anesthesia and ultrasound-guided out-of-plane puncture, a 20-gauge arterial catheter was successfully placed on the first attempt (puncture-to-placement time: 25–30 s). Catheters remained in place for 24–48 h. Waveforms were clear, stable, and unaffected by wrist motion. Invasive pressures matched non-invasive measurements at concurrent time points—though formal agreement analysis was not performed. After removal, puncture sites healed without complications (no bleeding, hematoma, infection, nerve injury, radial artery occlusion, or hand ischemia). One patient reported mild soreness (VAS = 1).

**Conclusion:**

Distal radial artery cannulation in the Hegu region may be a feasible and well-tolerated alternative for perioperative invasive arterial pressure monitoring when conventional proximal radial artery access is unavailable or undesirable. Potential advantages include a puncture site away from the wrist joint, stable catheter fixation, good patient tolerance, and preserved collateral circulation.

## Introduction

Continuous and accurate arterial pressure monitoring is an essential component of perioperative hemodynamic management, particularly during major surgery, emergency trauma surgery, hemodynamic instability, and clinical scenarios requiring precise fluid therapy or titration of vasoactive medications. Compared with intermittent non-invasive blood pressure monitoring, an arterial catheter provides a continuous pressure waveform and real-time changes in arterial pressure and is therefore widely used in anesthesiology and critical care (1, 2).

The radial artery is the most commonly used arterial access site because it is superficial, easy to palpate, and associated with a relatively low rate of complications (3, 4). Nevertheless, the conventional proximal radial puncture site is usually located near the wrist crease and close to the wrist joint. Intraoperative positioning, wrist flexion or extension, postoperative limb movement, and local dressing or compression may lead to catheter displacement, mechanical compression, waveform damping, or interruption of monitoring. In addition, proximal radial artery cannulation may still be complicated by hematoma, arterial spasm, infection, nerve injury, arteriovenous fistula, or radial artery occlusion (4–6).

**Figure 1:**
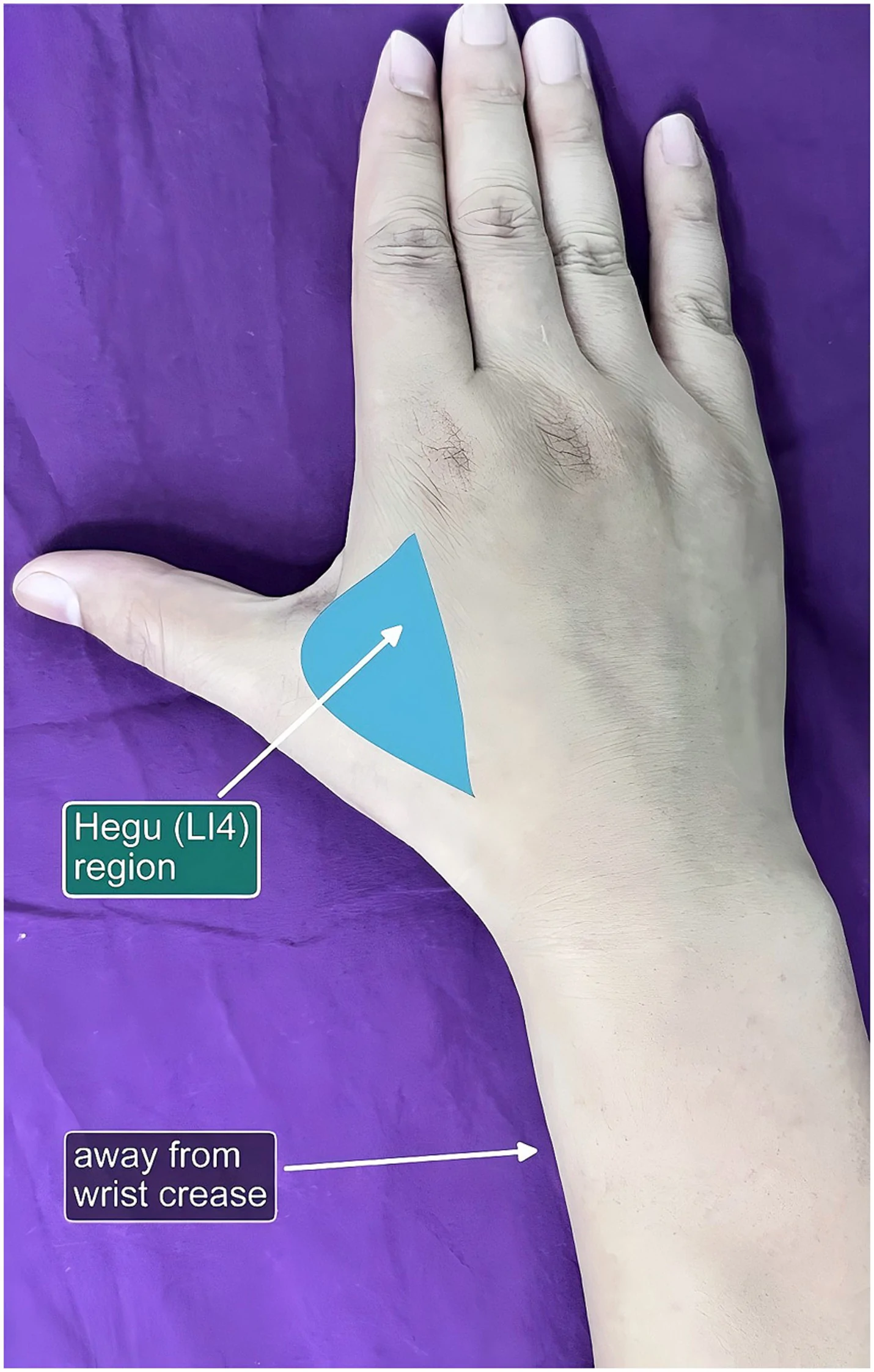
Surface localization for distal radial artery cannulation in the Hegu (LI4) region. Surface localization photograph showing the Hegu (LI4) region on the dorsum of the hand, away from the wrist crease.

Distal radial artery access, particularly in the anatomical snuffbox or the first web space around the Hegu (LI4) region, has attracted increasing attention in coronary angiography and percutaneous coronary intervention (5, 6). The Hegu point is located on the dorsum of the hand between the first and second metacarpals. In the adjacent region, the distal radial arterial pulse may be palpable, and the vessel is usually superficial. Because the puncture site is away from the wrist joint, catheter stability may be less affected by wrist motion (Figure 1). Moreover, if distal radial artery occlusion occurs, blood supply to the hand may still be maintained through the proximal radial artery, ulnar artery, and palmar arches in patients with adequate collateral circulation (5–8).

Evidence on distal radial artery access for perioperative invasive arterial pressure monitoring remains limited. Most available studies have focused on cardiovascular interventions rather than prolonged perioperative monitoring. Whether the puncture success, monitoring stability, and patient tolerance observed in interventional cardiology can be extrapolated to anesthetic monitoring is unclear. We therefore report two cases in which distal radial artery cannulation in the Hegu region was used for perioperative invasive arterial blood pressure monitoring when conventional proximal radial artery access was limited.

## Detailed case description

### Case 1: emergency right upper-limb replantation with unavailable conventional radial access

A 41-year-old woman weighing 65 kg, with an American Society of Anesthesiologists (ASA) physical status of III, was scheduled for emergency right upper-limb replantation after traumatic amputation caused by a machine accident. On arrival in the operating room, she was conscious and able to cooperate with the examination. Baseline vital signs were as follows: non-invasive blood pressure, 125/75 mmHg; heart rate, 92 beats/min; respiratory rate, 20 breaths/min; and peripheral oxygen saturation, 97% while breathing room air.

The right upper limb was severely injured and was unsuitable for arterial puncture. The left wrist had traumatic abrasions and swelling, and an intravenous infusion line had already been placed in the region. Conventional proximal radial artery cannulation was therefore considered relatively high risk. After ultrasound assessment of the left palmar collateral circulation, the left distal radial artery in the Hegu region was selected for invasive arterial pressure monitoring.

**Figure 2:**
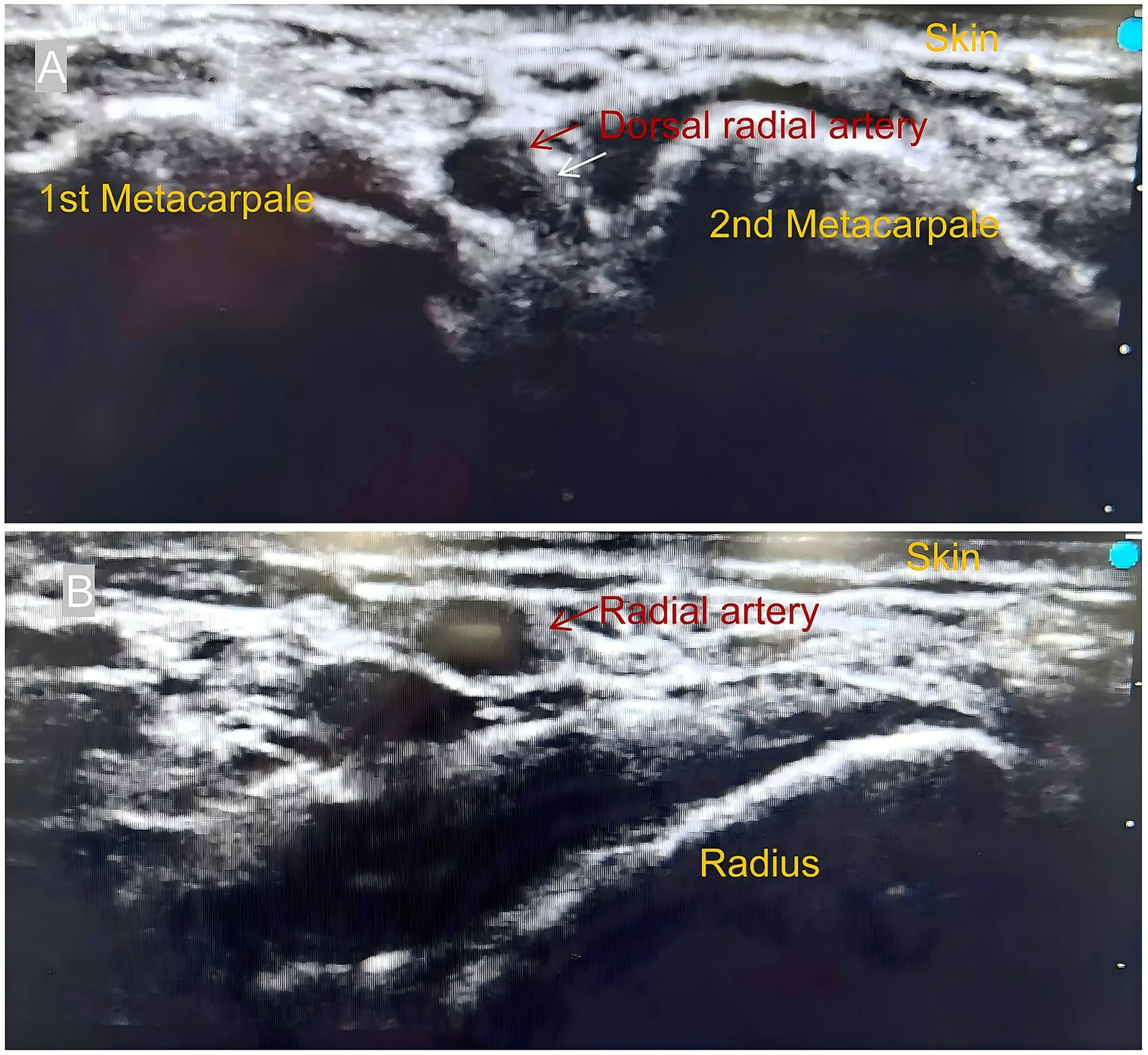
Comparison of ultrasound images of the distal radial artery in the Hegu region and the wrist radial artery. **(A)** The ultrasound images of the distal radial artery in the Hegu region; **(B)** The ultrasound images of the wrist radial artery.

After sterile skin preparation and draping, local infiltration anesthesia was performed with 2% lidocaine. A 5 to 12 MHz high-frequency linear ultrasound probe was used to scan the left Hegu region. The course, diameter, and superficial soft-tissue relationships of the distal radial artery were identified, and major anatomical variations were excluded (Figure 2). Ultrasound-guided out-of-plane puncture was then performed. The probe was placed transversely over the target vessel, and the needle was inserted approximately 0.5 cm lateral to the midpoint of the probe. A 20-gauge arterial catheter was advanced at an initial angle of 30° to 45°. Under real-time ultrasound visualization, the hyperechoic needle tip was seen approaching the anterior arterial wall. After tenting and puncture of the arterial wall, pulsatile bright-red blood return was observed. The puncture angle was then reduced to approximately 15°, the needle was advanced smoothly for about 2 mm, and the arterial catheter was inserted successfully. After free arterial backflow and correct catheter position were confirmed, the catheter was connected to a pressure transducer system, flushed, zeroed, and fixed with a transparent dressing. The entire procedure from skin puncture to successful catheter placement took approximately 30 s. No immediate bleeding, hematoma, or nerve injury was observed.

**Figure 3:**
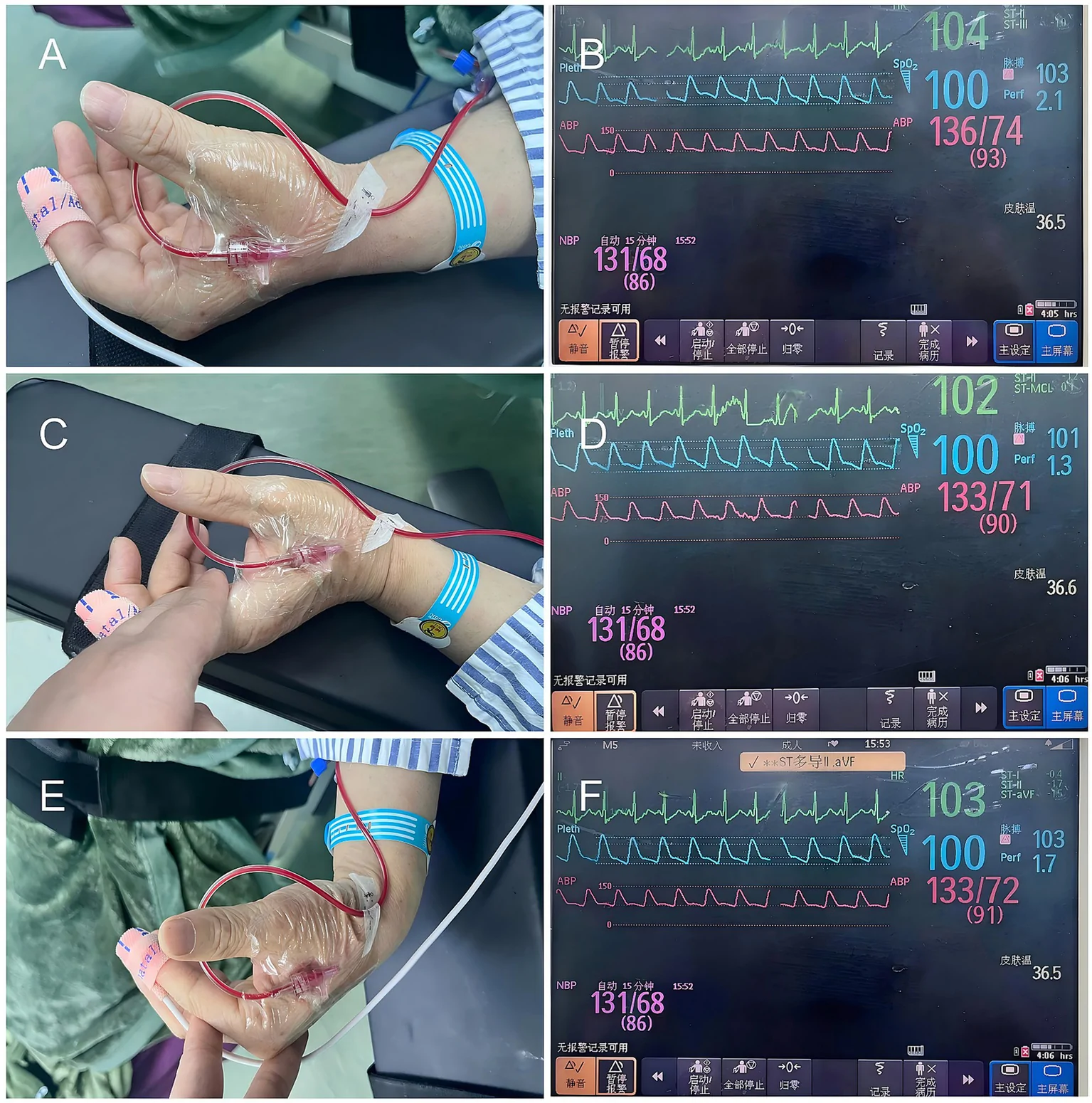
No significant changes were detected in the invasive arterial monitoring waveforms when the patient’s wrist changed positions. **(A)** Wrist at the rest position; **(B)** Arterial monitoring waveform when the wrist is at the rest position; **(C)** Wrist in the dorsiflexion position; **(D)** Arterial monitoring waveform when the wrist is in the dorsiflexion position; **(E)** Wrist in the palmar flexion position; **(F)** Arterial monitoring waveform when the wrist is in the palmar flexion position.

The catheter was retained for approximately 24 h. During monitoring, the invasive arterial pressure waveform remained clear and stable, without obvious distortion caused by wrist movement or changes in body position (Figure 3). The arterial blood pressure value is similar to the non-invasive blood pressure measurement on the same side, with the difference falling within a reasonable range. The catheter was removed on postoperative day 1. At 72 h after surgery, the puncture site had healed well, and no radial artery occlusion, infection, neurological impairment, or other puncture-related complication was observed. The patient reported no pain or discomfort, and sensory and motor function of the left hand were normal.

### Case 2: complex right-hand flap reconstruction with bilaterally limited conventional radial access

A 50-year-old man, 174 cm in height and 80 kg in weight, with an ASA physical status of II, was admitted for emergency surgery because of a right-hand crush injury with a skin defect and exposed bone. He had previously undergone wound debridement and vacuum sealing drainage at another hospital. Baseline vital signs were as follows: non-invasive blood pressure, 138/86 mmHg; heart rate, 89 beats/min; respiratory rate, 22 breaths/min; and peripheral oxygen saturation, 100% while breathing room air.

Further surgical repair was planned, including right-hand wound debridement, proximal interphalangeal joint internal fixation, free right anterolateral thigh flap transplantation, and split-thickness skin grafting. The expected operative duration was more than 5 h. Because of the complexity of the operation, the intensity of surgical stimulation, and the risk of perioperative hemodynamic fluctuation, continuous invasive arterial blood pressure monitoring was required to guide anesthetic management and fluid therapy.

The right upper limb was the primary operative field and had extensive soft-tissue and bone injury, precluding conventional radial artery cannulation. In addition, the left wrist had a history of recent radial artery puncture, with obvious subcutaneous ecchymosis at the puncture site, suggesting previous vascular and local soft-tissue injury. Repeated proximal radial puncture was considered to increase the risk of arterial spasm, hematoma, and postoperative vascular occlusion. The left distal radial artery in the Hegu region was therefore selected as the arterial monitoring route.

Before puncture, ultrasound was used to evaluate the course and diameter of the distal radial artery and the collateral circulation of the left palmar arch. Cannulation was performed using the same ultrasound-guided out-of-plane technique as in Case 1. The entire procedure took approximately 28 s and was not associated with significant bleeding, hematoma, or symptoms of nerve irritation.

During surgery, the invasive arterial pressure waveform was clear, continuous, and stable. The arterial blood pressure value is similar to the non-invasive blood pressure measurement on the same side, with the difference falling within a reasonable range. The real-time hemodynamic information provided by the arterial catheter supported intraoperative fluid management and maintenance of circulatory stability. The catheter was removed on postoperative day 2. The puncture site was dry, without a visible hematoma, swelling, or infection. The patient reported only mild soreness at the puncture site, with a visual analogue scale score of 1, and tolerated the procedure well. At 72 h after surgery, sensory and motor function of the left hand and wrist were normal, and no signs of hand ischemia or delayed complications were observed.

## Discussion

This report describes two patients in whom distal radial artery cannulation in the Hegu region was used for perioperative invasive arterial pressure monitoring when conventional proximal radial artery access was limited. Cannulation was successful on the first attempt in both patients, arterial pressure waveforms remained stable during monitoring, and no obvious puncture-related complication was detected during short-term follow-up. These cases suggest that the Hegu region distal radial artery may be a feasible alternative access route for invasive arterial pressure monitoring in selected clinical settings. The main clinical value of this approach lies in extending an access route originally used mainly in cardiovascular intervention to the perioperative hemodynamic monitoring setting.

### Anatomical and technical rationale

The puncture site used in this report was located in the Hegu region between the bases of the first and second metacarpals. In this region, the distal radial artery is relatively superficial and is supported by surrounding connective tissue. Compared with the conventional proximal radial approach at the wrist, this site is farther from the wrist joint and may therefore reduce the influence of wrist flexion, extension, rotation, and postoperative activity on catheter position and pressure waveform quality. In both patients, no obvious waveform distortion related to wrist movement was observed, which is consistent with the presumed advantage of this access site for catheter stability.

Another potential advantage is related to collateral perfusion. Distal radial artery occlusion, if it occurs, is expected to affect a more distal arterial segment than proximal radial artery occlusion. Because hand perfusion is supplied by both the radial and ulnar arteries through the superficial and deep palmar arches, severe hand ischemia may be less likely after distal radial artery occlusion when collateral circulation is adequate (9). However, this assumption does not eliminate the need for careful assessment. Patients with incomplete palmar arches, peripheral vascular disease, diabetes mellitus, previous vascular injury, or abnormal collateral circulation should be evaluated cautiously before this approach is selected.

### Extension from cardiovascular intervention to perioperative monitoring

Since distal radial artery access was introduced for coronary procedures, increasing evidence has accumulated in the cardiovascular field. Previous studies have suggested that, compared with the conventional proximal radial approach, distal radial access may reduce proximal radial artery occlusion, access-site bleeding, and some puncture-related complications (7). However, cardiovascular interventions usually involve short-term arterial access, whereas perioperative monitoring may require catheter retention for several hours to several days. During surgery and recovery, the patient may undergo changes in position, passive limb movement, and active wrist motion after awakening. Therefore, the safety and stability data from cardiovascular intervention cannot be directly assumed to apply to anesthetic monitoring.

In the present cases, catheter dwell time was 24 to 48 h, and the pressure waveform remained stable throughout monitoring. Wrist movement did not obviously affect the waveform. Although these observations are limited by the small number of patients, they suggest that the potential mechanical advantage of the Hegu region puncture site may persist during prolonged catheter retention.

### Procedural feasibility and the role of ultrasound guidance

Both cannulations were performed using ultrasound-guided out-of-plane puncture. Compared with palpation-guided puncture alone, ultrasound guidance offers several advantages. First, it allows preprocedural evaluation of vessel diameter, arterial course, anatomical variation, and ipsilateral palmar collateral circulation. Second, it provides real-time visualization of the relationship between the needle tip and the vessel wall, which may reduce repeated punctures, arterial spasm, and hematoma. Third, it is particularly useful in patients with obesity, edema, weak arterial pulsation, shock, or hypotension.

In cardiovascular practice, distal radial artery puncture is often performed with palpation guidance. In anesthetic practice, however, patient factors such as trauma, edema, low blood pressure, or weak pulses are common. In the two cases reported here, ultrasound-guided puncture was completed within approximately 25 to 30 s. This short procedure time may reflect the relatively fixed arterial course and recognizable surface landmarks in the Hegu region, as well as operator experience. During wider clinical adoption, routine ultrasound guidance may help shorten the learning curve and improve first-attempt success. Previous work has shown that ultrasound guidance can markedly improve first-attempt success for arterial puncture compared with traditional palpation guidance (10).

### Potential clinical scenarios

The two cases represent common situations in which conventional arterial access is limited in anesthetic practice. The first involved emergency upper-limb trauma in which the injured limb could not be used for arterial cannulation and the contralateral wrist was locally injured. The second involved complex hand reconstruction in which the operative limb was unavailable and the contralateral wrist had evidence of recent puncture-related injury. These scenarios suggest that Hegu region distal radial artery cannulation may address an unmet need in patients who require continuous arterial pressure monitoring but have limited access options.

Because the puncture site is away from the wrist joint, this approach may also be useful when wrist motion, surgical positioning, or postoperative activity is expected to compromise a conventional proximal radial catheter. In both cases, the arterial waveform remained stable despite the possibility of wrist movement and perioperative repositioning. This feature may be clinically relevant during long procedures and postoperative monitoring.

### Safety considerations

No bleeding, hematoma, infection, neurological deficit, arterial occlusion, or hand ischemia was observed in either patient during short-term follow-up. Patient tolerance was good, and only one patient reported mild puncture-site soreness. These findings are encouraging but should be interpreted cautiously. The absence of complications in two patients does not establish safety. Before this approach is used routinely, operators should evaluate collateral circulation, avoid repeated puncture, monitor puncture-site complications, and consider post-procedural assessment for asymptomatic radial artery occlusion when clinically indicated.

### Limitations

This report has several limitations. First, it includes only two patients. Although the cases represent clinically relevant difficult-access scenarios, they cannot provide robust evidence on success rate, monitoring accuracy, or complication risks. Prospective comparative studies are required to evaluate Hegu-region distal radial artery cannulation against conventional proximal radial artery cannulation in terms of first-attempt success, procedure time, waveform quality, catheter stability, patient comfort, and short- and long-term complications. Second, follow-up was limited to 72 h. Longer follow-up is needed to detect delayed vascular or neurological complications, including asymptomatic radial artery occlusion and occult arteriovenous fistulas. Third, high-risk groups, such as older patients, patients with coagulopathy, diabetes mellitus, peripheral vascular disease, or incomplete palmar collateral circulation were not represented. Fourth, both procedures were performed by an experienced anesthesiologist, and the reproducibility of the technique among operators with different levels of experience remains to be evaluated. Finally, the comparison between invasive and non-invasive blood pressure values was descriptive and cannot replace a formal agreement or accuracy analysis.

## Conclusion

Distal radial artery cannulation in the Hegu (LI4) region may provide a feasible alternative for perioperative invasive arterial blood pressure monitoring when conventional proximal radial artery access is unavailable or undesirable. Compared with the conventional wrist approach, this technique may offer a puncture site away from the wrist joint, stable pressure waveforms, good patient tolerance, and a favorable collateral circulation basis. However, current evidence remains preliminary. Larger prospective studies are needed to define success rates, monitoring accuracy, catheter stability, patient comfort, and complication profiles before this technique can be recommended for routine use.

## Data Availability

The original contributions presented in the study are included in the article/supplementary material, further inquiries can be directed to the corresponding author.
